# Computer game-based upper extremity training in the home environment in stroke persons: a single subject design

**DOI:** 10.1186/1743-0003-11-35

**Published:** 2014-03-13

**Authors:** Angelique Slijper, Karin E Svensson, Per Backlund, Henrik Engström, Katharina Stibrant Sunnerhagen

**Affiliations:** 1 Rehabilitation Medicine, The Institute of Neuroscience and Physiology, Sahlgrenska Academy at the University of Gothenburg, Per Dubbsgatan 14 3rd floor, SU/Sahlgrenska, Göteborg SE-413 45, Sweden; 2 Department of Occupational Therapy and Physiotherapy, Skaraborg Hospital Skövde, Skövde, Sweden; 3 School of Informatics, University of Skövde, Skövde, Sweden

**Keywords:** **S**troke, Upper extremity, Community living, Home-based rehabilitation, Computer games, Neurological rehabilitation

## Abstract

**Background:**

The objective of the present study was to assess whether computer game-based training in the home setting in the late phase after stroke could improve upper extremity motor function.

**Methods:**

Twelve subjects with prior stroke were recruited; 11 completed the study.

**Design:**

The study had a single subject design; there was a baseline test (A1), a during intervention test (B) once a week, a post-test (A2) measured directly after the treatment phase, plus a follow-up (C) 16–18 weeks after the treatment phase. Information on motor function (Fugl-Meyer), grip force (Grippit^R^) and arm function in activity (ARAT, ABILHAND) was gathered at A1, A2 and C. During B, only Fugl-Meyer and ARAT were measured. The intervention comprised five weeks of game-based computer training in the home environment. All games were designed to be controlled by either the affected arm alone or by both arms. Conventional formulae were used to calculate the mean, median and standard deviations. Wilcoxon’s signed rank test was used for tests of dependent samples. Continuous data were analyzed by methods for repeated measures and ordinal data were analyzed by methods for ordered multinomial data using cumulative logistic models. A p-value of < 0.05 was considered statistically significant.

**Results:**

Six females and five males, participated in the study with an average age of 58 years (range 26–66). FMA-UE A-D (motor function), ARAT, the maximal grip force and the mean grip force on the affected side show significant improvements at post-test and follow-up compared to baseline. No significant correlation was found between the amount of game time and changes in the outcomes investigated in this study.

**Conclusion:**

The results indicate that computer game-based training could be a promising approach to improve upper extremity function in the late phase after stroke, since in this study, changes were achieved in motor function and activity capacity.

## Introduction

Stroke is a leading cause of disability and is often associated with impaired motor function. Although most stroke survivors regain the ability to walk, many fail to regain functional use of their impaired upper extremity (UE) [1]. At six months, it is reported that only 11.6% of patients had achieved complete functional recovery, while dexterity in the paretic arm was found in 38% [2]. The impairment limits an individual’s ability to perform various activities of daily living (disability) and affects participation in everyday life situations [3].

After discharge from the stroke unit, there is still room for improvement that can be achieved with rehabilitation [4]. As rehabilitation is time-consuming, demanding and often tedious for the patient, it seems to be important to sustain the motivation for training [5]. Being given the possibility to choose where, when, what and how much rehabilitation are ways to stimulate empowerment, which may be a way to keep motivation. There is an awareness that self-controlled practice seems to enhance motor learning [6].

The main general recommendation to promote UE motor recovery after stroke is to focus on high-intensity; repetitive task-specific practice with feedback on performance, however there is no specific state of the art for training [7]. One suggestion is bilateral arm training, which is simultaneous active movement of the paretic and the non-affected arm [8-10]. Today methods are used and tested that combine computer technology and games for UE rehabilitation [11-14]. The game component is used as a motivational factor in terms of enjoyment, challenge and feedback [15]. The concept of using games for purposes other than entertainment is referred to as serious games [16,17]. There are serious games that have been specially developed for recovery and rehabilitation [11].

There is evidence that guided home rehabilitation prevents patients from deteriorating in their ability to undertake activities of daily living [18]. When guided home rehabilitation has a technology that allows patients to perform training with minimal therapist time the patients have the opportunity to practice more often, which may lead to functional improvement [19-21]. An advantage of rehabilitation in the home, is that it saves time and transportation.

There is a need to provide methods for UE rehabilitation overall. Considering that high intensive repetitive practice, bilateral arm training, feedback, self-controlled practice and motivation are important factors, a solution could be computer game-based training.

A benefit is if it can be used in the home environment. The experiences from a pilot study using computer game-based training in home environment suggested that it could be an alternative [22].

The aim of this exploratory study was to investigate if there is an intervention effect of computer game-based training in the late phase after stroke on upper extremity motor function.

## Material and methods

The single subject design [23] was chosen for this exploratory trial since this design is sensitive to individual differences and the individual variations after the stroke are large. A convenience sample of 12 subjects with prior stroke was recruited from a rehabilitation clinic. Inclusion criteria were diagnosis of stroke at least six months prior to the study, affected motor function in the upper extremity, National Institutes of Health Stroke Scale [24] (NIHSS) < 15 at study start and a minimum age of 18 years. Exclusion criteria were other neurological disease, diagnosed dementia or epilepsy, joint problems or pain in the upper extremity or language difficulty that would affect the capacity to receive information about the training procedure.

Twelve subjects fulfilled the criteria and gave their written informed consent to participate. One subject dropped out due to medical complications before the intervention started. Due to the design of the trial (single subject) no data could be that person (only first assessment was available from the baseline).

Prior to the baseline test the subjects were given an introduction to the system at the clinic to see whether they were able to use the equipment. This was done by letting the subject try the game that required the least motor function for 15 minutes.

### Research design

The study had a single subject design [23]; a baseline (A1) phase, a phase during intervention (B), a post-test (A2), and a follow-up (C). After the assessments for the baseline (A1), the treatment phase (B) started and continued for five weeks. The post-intervention (A2) was measured directly after the treatment phase and a follow-up (C) assessment was made 16–18 weeks later with the same assessment procedure as used in the pre-intervention. For practical reasons, since there were only 6 computers with games, 6 persons were able to train at a time. The study included 2 therapists, who both had participated in the pilot test and were familiar with the equipment. One of the therapists conducted all the assessment for 5 persons, the other therapist delivered the game based computer at home and coached the subject once a week during the treatment phase. Their roles were switched for the following 6 persons. Details on tests and timing are presented below.

### Computer equipment

**Figure 1:**
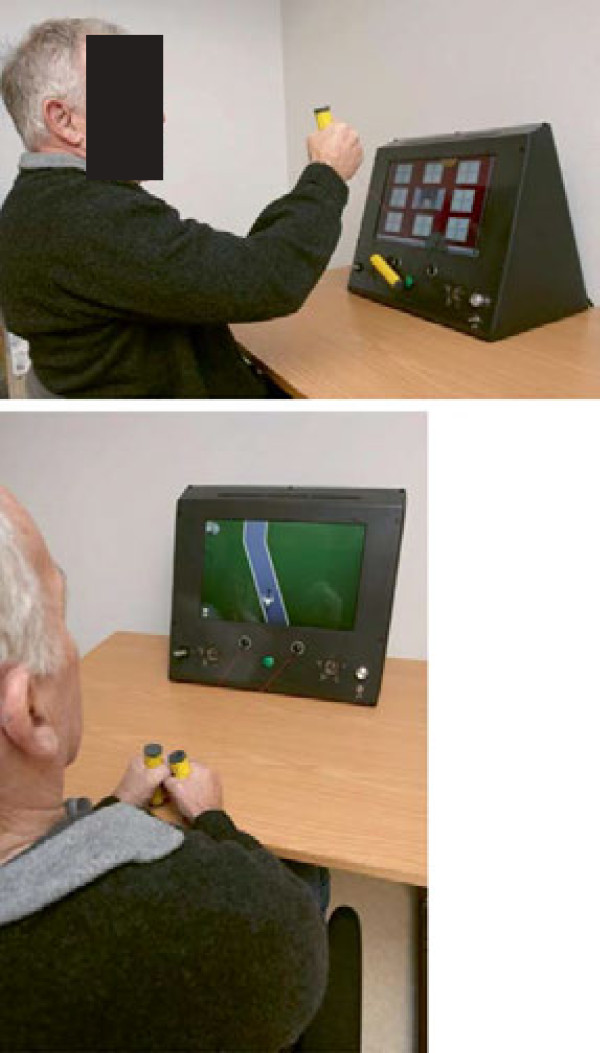
Game console in action.

The training was performed using a game console controlled by arm movements. The game console was based on a laptop computer and had two handles attached to it by strings. The handles were held with a transverse cylindrical grip and were connected to a mechanism that registered the position and movement of the arm. The game console (Figure 1) had a simple interface composed of an on/off button, a volume control, a game reset button and a USB plug for personal activity logs. The user log system captured rich information about a player’s activities in the system. Hand position in X, Y and Z axis was logged 25 times per second. Other system events such as which game was played, all game events and scores were also logged. The game console was programmed according to the affected side of the subject.

All games were designed to be controlled by either the affected arm alone or by both arms. There were 15 games in the library, games inspired by classic video games (such as Breakout, Snake and Puzzle) and newly developed ones (Table 1). The games were tailored to the intended users in terms of speed, challenge, toned down colour schemes and sounds. Prior to each game, verbal and visual instructions were given in the form of an animated film.

**Table 1:** The games used in the study, interaction model, skills and introduction timing

**Name**	**Game element**	**Basic interaction model**	**Skills/challenges**	**Day introduced**
Recycling	Kick empty cans of a ramp, avoid to hit the gnomes	One-hand, trigger	Timing	0
Bike ride	Run the bicycle from start to goal avoiding various obstacles	Two hand, synchronous 1D-move	Planning, precision, timing	1
Water war	Throw a water balloon on a kid, avoid the father	One-hand 2D-move	Precision, speed	3
Autumn shooting range	Hit the moose that passes by, avoid hitting the gnomes	One-hand, trigger	Timing	5
Breakout	Hit all bricks with a ball which should be stopped from dropping out	One-hand, 1D-move	Planning, precision, timing	7
Trombone	Hit the notes with the trombone.	One-hand, 1D-move	Precision, timing	8
Rowing	Row as fast as possible compete with previous result	Two hand, synchronous 1D-move	Speed	9
Summer shooting Range	Hit the moose by aiming in a horizontal direction. Avoid hitting the gnomes.	One-hand, 1D-move	Precision, speed	11
Puzzle bubble	Pop bubbles by pairing similar colors. If three bubbles with the same color are connected the pop.	Two-hand, 1D move and trigger	Planning, precision	12
Hurdles	Run from start to goal and jump over hurdles.	One-hand 2D-move	Timing	13
Boxing	Hit the opponent before he hits you.	Two-hand, alternate 1D move	Speed, timing	15
Pop-bubble	Pop all bubbles except the red ones.	One-hand 2D-move	Precision	17
Slingshot	Use the slingshot to aim and shoot at can-pyramids.	Two-hand, 2D-move	Precision, (planning), speed	20
Snake	Steer a snake to eat apples (!) avoid eating its tail.	One-hand. 2D move	Planning, timing	22
Winter shooting range	Hit the moose by aiming in a horizontal and vertical direction. Avoid hitting the gnomes.	One-hand 2D-move	Precision, speed	24
Paint	None	Two-hand 3D-move	-	26
Dash	Run as fast as possible from start to goal. Compete with previous result.	Two-hand, alternate 1D move	Speed	28

All games started at an easy level and difficulty increased with improved results demanding greater speed, precision and range of motion. The different games were delivered a few at the time throughout the experiment. All players received a very easy game called Recycling first in order to learn initial operations. As the number of games increased the participants were allowed to choose their own games. Visual and auditory feedback on how the games succeeded was given by the game console during game time and after a game was completed. A five-minute break was scheduled after 15 minutes of play.

### Outcome measures

#### Game time

The time that each console had been used was recorded.

#### Body function

##### Grip force

To record the handgrip force, the peak maximum grip force for each hand and the mean value over 10 s, as measured by the Grippit^R^, was evaluated in a standardized manner. The grip force was compared to age- and sex-matched reference values [25]. This test has been shown to have good reliability [26,27].

The motor function was assessed with the Fugl-Meyer Assessment of sensorimotor function, using the upper extremity part (FMA-UE) [28]. The FMA-UE, which is composed of 33 items related to movements of the proximal and distal parts of the UEs. Fugl-Meyer is one of the most frequently measures used in trials to evaluate UE. Several studies evaluating the psychometric properties of the FMA-UE in people with stroke have demonstrated satisfactory reliability, validity and responsiveness [29,30]. The items of the FMA-UE are mainly scored on a three-point scale, from 0 to 2 and the total score ranges from 0 to 66. The assessment was performed in a standardized manner. The Fugl-Meyer scale assesses motor function (A-D), sensation (H) and passive joint motion and joint pain (J).

#### Activity capacity

The Action Research Arm Test (ARAT) is a standardized ordinal scale designed to assess UE disability through the assessment of four basic movements: primary grasp, grip, pinch, and gross movements of flexion and extension at the elbow and shoulder [31]. The reliability, validity and responsiveness of the ARAT for people with stroke have been established [31]. The ARAT comprises 19 test of arm function in a standardized approach. Each test is graded on a four-point scale, from 0 (unable to complete any part of the hand or arm movement components) to 3 (normal performance), yielding a maximum for the test of 57. The test was performed in a standardized manner with a dedicated set-up of the test equipment. For example in the category grip the subject is asked to pour the water from one cup to another cup and have to manage that within 5 seconds without spilling and without compensating movements like later flexion in the trunk.

#### Activity performance

ABILHAND measures the patient’s perceived difficulty in performing everyday manual activities [32]. Recent studies from other researchers have demonstrated satisfactory reliability, validity and responsiveness [33,34]. ABILHAND is an inventory of manual activities that the patient is asked to judge on a three-level scale: 0 (impossible), 1 (difficult) and 2 (easy). The test explores both unimanual and bimanual activities done without human or technical help. For each question the patient provided his/her feeling of difficulty irrespective of the limb(s) actually used to perform the activity. For example is the subject asked to judge “cutting one’s nails, fastening the zipper of a jacket”. According to the manual, activities not attempted in the last three months were encoded as missing responses. The Swedish version was used [35] and the responses were entered into the program of http://www.rehab-scales.org/abilhand.html and transformed into logits [32].

### Procedures regarding data gathering and intervention

Fugl-Meyer, Grippit^R^, ARAT and ABILHAND was gathered at baseline (A1), post-test (A2) and follow-up (C). During the intervention (B), only Fugl-Meyer and ARAT were measured.

The intervention comprised five weeks of game-based computer training in the home environment. A game console was delivered to the participants’ home by a member of the development team and one of the therapists. The place where the subject intended to place the game console for playing was reviewed for ergonomics and suggestions were given. The subjects went through the game console once more and were given a short manual in order to be able to handle the game console on their own. They were told to play as much as they liked during this five week period; no specific recommendations were made as to how much they should play. During this five week period, the subjects went once a week (four times) to the clinic to see the therapist for testing and the therapist for coaching. On these occasions, which also included a game session, the coaching therapist checked that movements were performed in an optimal manner without risk of injury, that the subjects could manage the system and that they understood how to play the games.

### Ethics and statistics

The study was approved by the Regional Ethical Review Board in Gothenburg (Dnr 643–07). All subjects gave their written informed consent. Statistical analyses were performed using PASW v.18 (SPSS). Conventional formulae were used to calculate the mean, median and standard deviations. Wilcoxon’s signed rank test was used for tests of dependent samples.

Continuous data were analyzed by methods for repeated measures using the SAS-procedure PROC MIXED. Means of measurements during intervention, post-test and follow-up respectively were compared to measurements of data from baseline. The deviations from baseline were tested by t-tests. In order to get more symmetric distribution log transformed data were used in the calculations. Ordinal data were analyzed by methods for ordered multinomial data using cumulative logistic models. The SAS-procedure PROC GLIMMIX was used. Deviations from baseline as above were tested. A p-value of <0.05 was considered statistically significant.

## Results

The eleven subjects in the study (six females and five males), had an average age of 58 years (range 26–66). All subjects were in the so called “chronic” stage after stroke; the median time since stroke onset was 11 months. Five subjects had an ischemic and six had a haemorrhagic stroke. Six subjects were impaired in the dominant hand (Table 2). At discharge from hospital, the Modified Rankin Scale (MRS) was 3 for six subjects and 4 for five subjects. All subjects lived (in the community) in their own homes, three were single, and two had minor children in the home. Eight subjects were retired or on disability pension, two subjects worked part-time and one subject worked full-time.

**Table 2:** Subject characteristics at the time of the study

**n**	**Sex**	**Age (years)**	**Time since stroke (months)**	**Hemiplegia (left/right)**	**Hand dominance**
1	M	66	20	Right	Right
2	F	58	10	Left	Left
3	F	60	6	Left	Right
4	M	48	7	Right	Right
5	M	65	42	Right	Right
6	M	26	8	Left	Right
7	F	64	11	Left	Right
8	F	31	13	Left	Right
9	M	58	16	Right	Right
10	F	57	11	Left	Right
11	F	53	16	Right	Right

The mean time at the game-based computer was 1070 min (range 267–4727). The mean time of days of play out of a maximum of 35 was 24.5 (range 19–35) (Table 3). One subject completed the protocol but did not return for the follow-up due to a medical problem. One person (n 8) was away for one week during the intervention.

**Table 3:** Time with game console

	**Time with game console (minutes)**	**Days of playing out of max 35**	**Number of sessions**
**1**	1012	22	43
**2**	1283	27	83
**3**	4727	35	170
**4**	267	26	28
**5**	545	23	39
**6**	464	25	36
**7**	862	24	52
**8**	316	19	22
**9**	704	19	46
**10**	762	25	49
**11**	831	24	49

**Figure 2:**
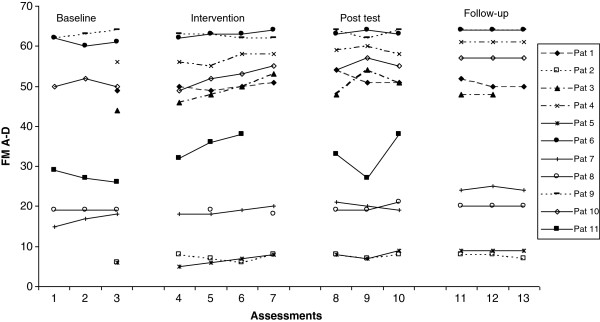
**Fugl-Meyer motor function changes during all tests, shown for all participants.** The number below indicates the test-occasion. As can be seen, due to administrative reasons, some participants were only tested once prior to intervention.

**Figure 3:**
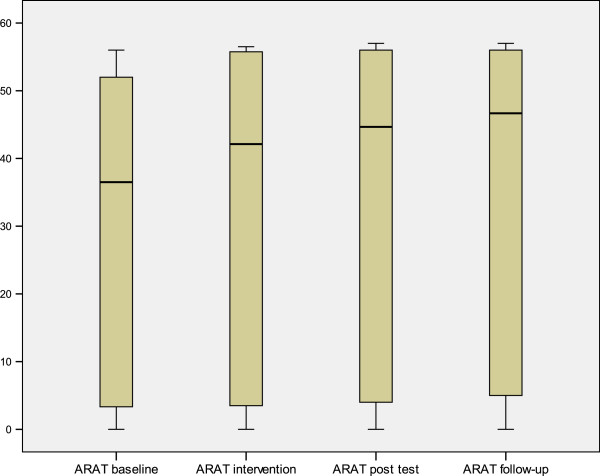
The box-plots are showing the median (thick line), the inter-quartiles and whiskers (smallest and largest value) of the Action Research Arm Test (ARAT) and illustrate the improvement with time.

The median value and range for Fugl-Meyer Assessment upper extremity (FMA-UE), ARAT, ABILHAND and Grippit^R^ for all subjects during each phase are shown in Table 4. An improvement in motor function was noted in the affected upper limb. FMA-UE A-D (motor function) (Figure 2), showed significant improvements in upper extremity function between baseline (A1) and post-test (A2) (0.005) as well as at follow-up (<.0001). The changes in ARAT (Figure 3) improved significantly (<.0001) both at post-test (A2) and at the four-month follow-up compared to baseline.

**Table 4:** The median value and range for the different assessments

	**Pre-intervention**	**Intervention**	**Post-intervention**	**Follow up**
	**Median**	**Median**	**Median**	**Median**
Fugl-Meyer A-D	44 (6–63)	49 (6.50-63)	51 (7.67-63.33)	49 (7.67-64)
ARAT	26 (0–56)	34 (0–56.2)	37 (0–57)	47 (0–57)
ABILHAND	0.36 (−.26-2.64)		0.86 (−.78-3.6)	1.13 (−.47-4.69)
Maximal grip A (% of normal)	27 (2–72)		26 (5–80)	39 (12–81)
Maximal grip NA	78 (59–118)		78 (56–110)	89.50 (59–113)

A = Affected.

NA = Non affected.

Median of measurements post-test and follow-up respectively were compared to measurements of data from baseline, and showed significant improvement (0.005- < .0001) in max Grip force on the affected side as well as the mean Grip force. This was not the case for the un-affected side. The ratio between affected and non-affected maximal grip force (% of reference value) showed a mean value of 0.41 at baseline (A1), 0.44 at post-test (A2) and 0.47 at follow-up (C).

No significant correlation was found between the amount of game time and changes in the outcomes investigated in this study.

## Discussion

The objective of the present study was to assess whether computer game-based training in the late phase after stroke could improve upper extremity motor function. The intervention improved upper extremity motor function and also improved activity capacity, and this improvement was maintained at follow-up. Motor function assessed by the Fugl-Meyer scale has been suggested to have a minimal detectable change for FMA-UE of 5.2 points [29] and according to another author, the minimal clinical important difference is 10 points [36]. In the present study, 7 points between pre-intervention and post-intervention and 5 points between pre-intervention and follow-up. Having a statistically significant improvement in FMA-UE and at the same time no clinical difference has been seen also in other studies [37,38] .

There was a statistically significant improvement on the ARAT. According to the literature, there must be a difference of at least six points to define a minimal clinical important difference [39]. In this study, the median difference was 11 points between baseline and post-test and 21 points between baseline and follow-up, which shows a clinical definable difference. In the present case, ARAT showed a clinical important difference and FMA-UE did not. These results support the findings of other studies [29,40], suggesting that ARAT has the highest responsiveness in a comparison with FMA-UE.

The perceived number of problems as assessed with ABILHAND showed no difference. This is not surprising since the actual performance is not necessarily reflected in perceived functions. Michielsen et al. [41] reported that function and capacity must reach a certain threshold level before actual performance also starts to increase.

The participants in this study were interested in the games. The game factors, such as challenges and scores, had an important impact. Data about player’s behavior and interest in the games were collected through log files, observations and interviews. The results reveal a positive attitude towards the games as well as a substantial time spent on playing the games. Results regarding the attitudes were analyzed in detail in the pilot study and have been presented in detail in Alklind Taylor et al. [42]. The participants seemed to develop a taste for certain games as their favorites. Interestingly, remakes of classic games such as Breakout and Puzzle bubble were the most popular.

Feedback through the games repeatedly encouraged the users to improve their performance. Not only arm movements but also concentrating on the games was important. The player was required to attend, comprehend, recall and plan and execute appropriate responses to the visual and auditory cues provided. The challenging component of the games could be at the expense of carrying out tasks correctly but is required to retain motivation.

### Study limitations

It is reasonable to suggest that the ultimate aim is to promote restoration of function to the point at which the stroke patient can use the arm in everyday tasks. The games in this computer-based training were not specifically designed to increase the use of the arm in everyday tasks, and changes in activities are thus not to be taken for granted. As always in single subject design, the subject serves as their own control. The single subject design only makes it possible to assess whether there is a change achieved by an intervention. The results are similar to another small study where similar outcome measures were used [43]. A strength in this study is that assessments were made that cover different domains of the ICF. The selections of assessments are seen by others as good [44,45].

## Conclusion

The results indicate that computer game-based training appears to be a promising approach to improving upper extremity function in the late phase after stroke.

## Consent

Written informed consent was obtained from the patients for the publication of this report and any accompanying images.

## Competing interests

All authors state that they have no competing interests.

## Authors’ contribution

AS and KES carried out the study and wrote the first draft. PB and HE constructed the ELINOR, contents of the games etc and were responsible for extracting data from the computer. KSS designed the study and supervised AS and KES in the process, analyses and writing. All authors contributed in the writing process. All authors read and approved the final manuscript.
